# Comprehensive Degrees

**Published:** 1885-06

**Authors:** C. E. H. Phillips

**Affiliations:** New York


### COMPREHENSIVE DEGREES.

BY C. E. H. PHILLIPS, D. D. S., NEW YORK.

Throughout the world the degree of M. D. affixed to a name immediately conveys the understanding that its possessor is a doctor of medicine. There being but the one degree, it cannot be mistaken.

How very different is it with the practitioner of dentistry. Four neighbors may, side by side, practice this specialty, and each endeavor by a different degree to acquaint the laity with his calling. Is it then to be wondered at if the public eye is sorely puzzled in scanning such titles as D. D. S., M. D. S., L. D. S. and D. M. D., each indicating the possessor to be a practitioner of dentistry?

If a higher standard is represented by any one of these four degrees, would it not be well for colleges and State examining boards to insist upon meeting all requirements, and confer only that one degree, thus obviating perplexity and misunderstanding? The degree of dentistry should be as comprehensive to the general public as that of medicine.

